# Predicting the Willingness to Engage in Non-Consensual Forwarding of Sexts: The Role of Pornography and Instrumental Notions of Sex

**DOI:** 10.1007/s10508-019-01580-2

**Published:** 2020-01-31

**Authors:** Johanna M. F. van Oosten, Laura Vandenbosch

**Affiliations:** 1 The Amsterdam School of Communication Research (ASCoR), University of Amsterdam, Nieuwe Achtergracht 166, Postbus 15791, 1001 NG Amsterdam, The Netherlands; 2 School for Mass Communication Research, KU Leuven, Leuven, Belgium

**Keywords:** Sexting, Pornography, Instrumental attitudes, Adolescence, Emerging adults, Online harassment

## Abstract

Although non-consensual forwarding of sexts (NCFS) is an important type of online sexual harassment behavior, the predictors of this behavior are currently understudied. The present study aimed to fill this gap by investigating online pornography use as a predictor of adolescents’ and emerging adults’ willingness to engage in NCFS in different contexts (i.e., forwarding a sexually explicit picture of a dating partner, relationship partner, friend, stranger or ex-partner). Based on previous literature on the role of pornography in the prediction of sexual harassment, we hypothesized that this relationship would depend on individuals’ prior endorsement of sexual stereotypical attitudes (i.e., instrumental attitudes toward sex). We further investigated whether this would differ for adolescent and young adult males and females. We used data from a two-wave short-term (2 months between waves) longitudinal survey among 1947 participants (aged 13–25 years). Results from cross-lagged autoregressive latent SEM models showed that pornography use significantly predicted a higher willingness to forward sexts from a stranger, but mostly among adolescent boys (aged 13–17) with high levels of instrumental attitudes toward sex.

**Electronic supplementary material:**

The online version of this article (10.1007/s10508-019-01580-2) contains supplementary material, which is available to authorized users.

## Introduction

With the rise of digital technologies, young people have discovered new ways to explore their sexualities through the production of sexual media content, for instance, through sexting (Hasinoff, 2013). Sexting has been defined as “the sending, receiving, or forwarding of sexually explicit messages, images, or photos to others through electronic means, primarily between cellular phones” (Klettke, Hallford, & Mellor, 2014, p. 45). Sexting often occurs between two people in a romantic relationship and has become an increasingly common aspect of young people’s sexual exploration (Bianchi, Morelli, Baiocco, & Chirumbolo, 2016; Levine, 2013). At the same time, these developments have given rise to societal concerns about the non-consensual distribution of such sexual content. Sexting is considered a sexual risk behavior precisely because of the potential of the material being dispersed among many people (e.g., Döring, 2014; Lippman & Campbell, 2014; Ringrose, Harvey, Gill, & Livingstone, 2013; Walker, Sanci, & Temple-Smith, 2013; Yeung, Horyniak, Vella, Hellard, & Lim, 2014).

About 1.1–6.3% of young adults have experienced sexually explicit content of themselves being forwarded without their consent (e.g., Gámez-Guadix, Almendros, Borrajo, & Calvete, 2015; Marganski & Melander, 2018), and higher levels of depression and anxiety and lower levels of self-esteem have been found among victims of such antisocial online sexual behaviors (Priebe & Svedin, 2012). Engaging in the non-consensual forwarding of sexts (NCFS) is considered a form of online harassment (Walker & Sleath, 2017) that seems to be related to other sexual harassment indicators. One study found that sharing of someone else’s sexts without his/her consent was weakly, but significantly, related to both dating violence perpetration and benevolent and hostile sexism (Morelli, Bianchi, Baiocco, Pezzuti, & Chirumbolo, 2016).

Given the associations between NCFS and sexual harassment and lower well-being, it is striking that research has hardly focused on the predictors of this anti-social online sexual behavior. One survey (AP-MTV, 2009) did look at the most common reasons for forwarding sexts, which included the assumption that others wanted to see it, to show off to one’s friends, as a joke, or out of boredom. Such peer-related motives for NCFS have also been found in qualitative research (Van Ouytsel, Van Gool, Walrave, Ponnet, & Peeters, 2017). Moreover, it has been argued that an increasingly sexualized media culture could influence young people’s antisocial online sexual behaviors (Chalfen 2009, 2010; Ringrose et al. 2013). Although scholars have started to investigate media influences on (antisocial) online sexual behaviors in general (Bianchi, Morelli, Baiocco, & Chirumbolo, 2017; Morelli, Bianchi, Baiocco, Pezzuti, & Chirumbolo, 2017; Van Ouytsel, Ponnet, & Walrave 2014), research has not yet considered the potential influence of highly sexualized and sexual stereotypical mass media on NCFS in particular.

Accordingly, the present longitudinal study aimed to investigate whether the exposure to online pornography would predict adolescents’ and emerging adults’ willingness to engage in NCFS. Moreover, in line with previous research and theoretical models attesting to the importance of taking individual differences into account when studying the effects of sexually explicit media use (e.g., 3AM, Wright, 2011; the Confluence Model, Malamuth, Addison, & Koss, 2000; Malamuth & Huppin, 2005; Vega & Malamuth, 2007), we investigated whether the relationship between pornography use and the willingness to engage in NCFS would be most pronounced for youth who hold stronger pre-existing instrumental notions about sex, and whether this would further depend on gender and age (i.e., adolescents versus young adults).

### The Willingness to Engage in Non-Consensual Forwarding of Sexts

Studies have shown that between 3 and 24% of adolescents (e.g., Fleschler Peskin et al., 2013; Mitchell, Finkelhor, Jones, & Wolak, 2012; Strassberg, Rullo, & Mackaronis, 2014; Wood, Barter, Stanley, Aghtaie, & Larkins, 2015), and about 23% of adults (Garcia et al., 2016) have ever engaged in NCFS. This suggests that the large majority of young people still refrain from engaging in such behavior, although social desirability bias may affect these numbers. As NCFS can be considered a form of sexual harassment (DeKeseredy & Schwartz, 2016; Reed, Tolman, & Ward, 2016; Walker & Sleath, 2017) or antisocial online sexual behavior, it may not be a behavior that most youth deliberately *intend* to engage in, or admit to have engaged in.

A substantial body of literature has studied sexual behavior, and in particular relatively rare sexual risk or taboo behavior, from a perspective of behavioral willingness (e.g., Gerrard, Gibbons, Houlihan, Stock, & Pomery, 2008; Gibbons, Gerrard, Blanton, & Russell, 1998; Gibbons, Gerrard, & McCoy, 1995). This perspective grew out of the observation that people do not always intend to engage in certain (sexual) risky or taboo behavior, but may be willing to engage in such behavior when the situation lends itself for it. This behavioral willingness, in turn, makes future engagement in the behavior more likely (Gerrard et al., 2008; Gibbons & Gerrard, 1995; Gibbons et al., 1995, 1998).

Behavioral willingness may thus be a particularly relevant construct to investigate in relation to online sexual harassment, and NCFS in particular. It is important to know to what extent adolescents and emerging adults are willing to engage in NCFS, and what predicts such willingness, as it is a likely indication for future engagement in NCFS. For these reasons, the present study focuses on the willingness to engage in NCFS as an outcome variable. Furthermore, research on NCFS has hardly distinguished between different contexts in which such behavior can occur. Previous research did show that the sharing of sexual content is more likely to occur in the context of strangers (Baumgartner, Valkenburg, & Peter, 2010) and that the relationship between sexting and online victimization, and the anticipated consequences of such victimization, depend on the context in which it occurs (e.g., strangers, friends or dating partners, Bennett, Guran, Ramos, & Margolin, 2011; Gámez-Guadix et al., 2015). In the present study, we therefore distinguish between the willingness to engage in NCFS in different contexts, notably the context in which the victim is close to the perpetrator (i.e., friends, romantic relationship partners and dating partners) or not or no longer close to the perpetrator (i.e., strangers and ex-partners).

### Predicting the Willingness to Engage in Non-Consensual Forwarding of Sexts: The Role of Pornography Use

The lack of research on mass media content as a predictor of NCFS specifically is surprising given our knowledge on how mass media messages can predict harmful sexual behavior (Wright, 2011). More specifically, sexual harassment, both online and offline, has been associated with exposure to content that is said to promote such behavior, such as online pornography (e.g., Owens, Behun, Manning, & Reid, 2012; Stanley et al., 2016; Thompson & Morrison, 2013; see also Flood, 2009 for a review). However, these studies have not focused on (sexually explicit) media use in relation to (the willingness to engage in) non-consensual forwarding of sexts specifically.

Several theories have been used to explain the associations between sexual media use and sexual behavior (e.g., the 3A-Model; Wright, 2011; the Media Practice Model; Steele, 1999; Steele & Brown, 1995). According to these theories and models, pornography users take over behavioral scripts and attitudes from pornography and subsequently apply them to their daily life and own sexual behavior. Content analyses have shown that the sexual scripts typically portrayed in pornography reflect sexual harassment, such as women submitting to the desires of men, sexual coercion and/or violence, and objectifying or dehumanizing others (Cowan & Campbell, 1994; Cowan, Lee, Levy, & Snyder, 1988; Klaassen & Peter, 2015; Monk-Turner & Purcell, 1999; Seto, Maric, & Barbaree, 2001). Thus, pornography use may activate sexual harassment scripts that in turn increase one’s willingness to engage in (online) sexual harassment, such as the non-consensual forwarding of sexts. In fact, qualitative findings show that young people are aware that “the exchange of sexual messages and images could be informed by some of the abusive values and attitudes that underpin pornography” (Stanley et al., 2016, p. 21).

Moreover, sexual scripts from sexual media are more likely to be applied when it resonates with pre-existing experiences and beliefs (e.g., Leonhardt, Spencer, Butler, & Theobald, 2019; Wright, 2011). Research on antisocial attitudes and sexually aggressive behavior has repeatedly found that such attitudes and behaviors are more likely when there is a combination of pornography use and certain dispositions such as a noncommittal, impersonal orientation toward sex (e.g., Kingston, Malamuth, Fedoroff, & Marshall, 2009; Malamuth & Huppin, 2005; Malamuth et al., 2000; Vega & Malamuth, 2007). It has even been argued that “based on the importance of individual and cultural differences in moderating the relation between pornography and aggression, it is crucial that causal model theorists consider these moderating factors […]” (Kingston et al., 2009, p. 221). The relationship between pornography use and the willingness to engage in NCFS is thus expected to occur in interaction with pre-existing differential susceptibility variables.

### Individual Susceptibility: The Role of Instrumental Sexual Attitudes, Gender, and Age

One individual susceptibility characteristic that may be particularly relevant when studying the link between pornography use and the willingness to engage in NCFS is the endorsement of instrumental attitudes toward sex. Mass media, including pornography, have been promoting the idea that sex is “just a game,” and that the sole purpose of sex is to have pleasure and excitement without the need for love or intimacy (Brosius, Weaver, & Staab, 1993; Klaassen & Peter, 2015; Monk-Turner & Purcell, 1999; Ward & Rivadeneyra, 1999; Wright, 2009). In contrast to sexting, which often occurs within an intimate relationship (Bianchi et al., 2016; Levine, 2013), non-consensual forwarding of sexts, which consists of forwarding sexual images for one’s own fun and pleasure without considering the person in the picture, is a particularly instrumental type of sexual behavior. It can thus be expected that the portrayal of sex as purely physical and non-intimate in pornography resonates with pre-existing attitudes toward sex as purely physical, fun and exciting, which in turn increases individual’s willingness to engage in NCFS.

As described in the literature on the activation of sexual scripts by sexual media (Leonhardt et al., 2019; Wright, 2011), a double dose effect may follow where individual’s willingness to engage in instrumental sexual behaviors, such as forwarding a sexually explicit picture of someone without that person’s consent, may be reinforced more intensively among frequent pornography users with high levels of instrumental attitudes toward sex. Conversely, when pornography use conflicts with pre-existing sexual experience and beliefs obtained from other sources (such as parents and peers) its influence may be diminished (Leonhardt et al., 2019). We therefore hypothesized an interaction effect between pornography use and pre-existing instrumental attitudes toward sex, such that the relationship between pornography use and the willingness to engage in NCFS would occur mostly for youth with high levels of instrumental attitudes toward sex.

The manner in which pornography use resonates with pre-existing sexual attitudes and its relationship with NCFS may further depend on individual susceptibility based on gender and age. According to sexual socialization literature, males are generally taught a different type of sexuality than females. That is, males are taught to adopt a more recreational and non-committed view of sex, whereas females are taught to prefer relational sex and emotional intimacy (e.g., Masters, Casey, Wells, & Morrison, 2013). As a result, the content in pornography, as well as instrumental sexual attitudes and behaviors, is more likely to be congruent with the sexual socialization of males, thus leading to stronger effects among males. Relatedly, males have been shown to engage in more NCFS than females (e.g., Strassberg et al., 2014), to be more frequent pornography users, and, in some instance, seem to be more susceptible to the influence of pornography on sexual attitudes and behavior (Peter & Valkenburg, 2016). At the same time, mixed findings on gender differences have also been found, both for the engagement in NCFS (see Walker & Sleath, 2017 for a review) and the effects of pornography (Peter & Valkenburg, 2016). Moreover, within-gender differences in sexual scripts (Masters et al., 2013) suggest that gender, and gendered sexual socialization, may interact with people’s idiosyncratic notions about sex. In a similar vein, pornography use may interact with both instrumental attitudes toward sex and gender in its prediction of the willingness to engage in NCFS.

Furthermore, age may play an additional role in the relationship between the variables under study. In a previous study, the influence of pornography use on attitudes related to sexual harassment (i.e., the notion that when a woman says “no” to a sexual initiation she really means “yes”) has been shown to be stronger for adults compared to adolescents (Peter & Valkenburg, 2011). The authors explained this age difference as the result of the particular outcome variable in this study resonating more with the higher sexual experience among adults (Peter & Valkenburg, 2011). Although equal prevalence rates of NFCS have been found among adolescents (e.g., 24%, Wood et al., 2015) and adults (e.g., 23%, Garcia et al., 2016), a negative correlation between age and NCFS has been found among a sample of adolescents and emerging adults (Morelli et al., 2016), which suggests adolescents engage in NCFS more frequently than emerging adults.

Moreover, peer pressure is a main motivator for NCFS (AP-MTV, 2009; Van Ouytsel et al., 2017), and adolescents are known to be the most susceptible developmental group for peer influences (Steinberg & Monahan, 2007). Accordingly, NCFS may be particularly present in adolescence. Willingness to engage in NCFS may therefore resonate more with the lived experience of adolescents, resulting in a stronger influence of pornography use and sexual attitudes on such willingness among adolescents. Moreover, during adolescence, there is a co-occurring increase in pornography use, the endorsement of permissive or instrumental sexual attitudes and sexual behavior, particularly among adolescent boys (Doornwaard, Bickham, Rich, ter Bogt, & van den Eijnden, 2015). Given this simultaneous development, there may be a stronger resonance effect of pornography and instrumental attitudes toward sex among adolescents compared to young adults. Against this backdrop, it can thus be expected that the confluence of pornography and instrumental attitudes toward sex may differently affect the willingness to engage in NCFS for adolescent boys and girls and emerging adult males and females. We therefore also investigated whether the interaction effect of pornography use and instrumental attitudes on the willingness to engage in NCFS would further depend on gender and age.

## Method

### Participants and Procedure

Analyses were based on a two-wave longitudinal survey, with 2 months between waves, which was conducted among 2463 Dutch participants between April and June 2015 (response rate from Wave 1 to Wave 2 was 79%, resulting in a final sample of 1947). A time lag of 2 months was considered appropriate as it reflects the short-term changes in sexual behaviors that occur among youth (Kirby, Laris, & Rolleri, 2007), and has been used successfully in previous research on media effects (e.g., Gentile, Walsh, Ellison, Fox, & Cameron, 2004). Data from this survey have been used in previous studies of the authors (Vandenbosch & van Oosten, 2017; van Oosten & Vandenbosch, 2017). Half of the sample (51.7%, *N *=1007) consisted of adolescents (aged 13–17 years), and the other half (*N *=940) consisted of emerging adults (aged 18–25 years, cf. Arnett, 2000), and 48.2% of the sample was male. The majority of our sample (90.8%) had an exclusively heterosexual orientation. The sample was obtained from an existing online panel of a Dutch research agency that is representative of the Dutch population in terms of gender, SES, household size and residential area. After obtaining consent from the parents of the adolescent part of the sample, participants were invited to take part in a survey that asked about their media use and sexual behavior and attitudes. Informed consent of the participants was required before they could take part in the survey. Participants were asked to fill out the survey online and in private.

### Measures

#### Exposure to Online Pornography

Participants were asked how often in the previous 2 months they had intentionally looked at sexually explicit content (i.e., pornographic material, not nudity) on their computer, either online or offline (i.e., downloaded material) (cf. Peter & Valkenburg, 2010, 2011). Sexually explicit content was specified as (1) pictures in which people were having sex, and (2) movies in which people were having sex. Items about pictures and movies of clearly exposed genitals, often used in previous pornography use measures, were left out of the scale for the current analyses, given the conceptual overlap of such items with sexting. For both types of sexual content, the response categories were 1 (*several times a day*), 2 (*every day*), 3 (*several times a week*), 4 (*once a week*), 5 (1–3 *times a month*), 6 (*less than once a month*), and 7 (*never*). Items were recoded such that higher scores indicated more frequent use of online pornography. In both waves, the items formed a unidimensional scale (explained variance > 87%), and correlated strongly with each other, *r *> .74 (*M* = 1.87, SD = 1.45 in Wave 1; *M* = 1.90, SD = 1.45 in Wave 2).

#### Willingness to Engage in Non-Consensual Forwarding of Sexts

The measure of willingness to engage in NCFS was introduced by the following explanation (the adult version is shown between brackets): “Some boys (men) and girls (women) will send a picture of themselves to someone else via the internet or their smartphone in which they are naked or almost naked. Sometimes teens (people) forward such a picture to others without asking the person in the picture for permission.” Participants were then presented with the following scenario for the second part of the measure: “Imagine you received an image or video fragment on your phone or online that shows a person who is naked or almost naked. After you received it, you could forward this image or video to others without the sender being aware of it. Try to picture how likely it is that you would forward this image or video when it shows the following person: (1) the person that you are in a romantic relationship with; (2) the person that you are dating; (3) a friend; (4) someone you do not know; (5) your ex.” For each of the five persons that were mentioned the answer options ranged from 1 “very likely” to 7 “very unlikely”. Answers were recoded so that higher scores indicated a higher willingness to engage in NCFS. The five items were analyzed as separate outcome variables (see Table 1 for the properties of the willingness to engage in NCFS measure).

**Table 1:** Means and SDs of the willingness to engage in NCFS measures, for the total sample and the separate gender and age groups

	Total sample	Adolescent girls	Adolescent boys	Young adult women	Young adult men
	*M* (SD)	*M* (SD)	*M* (SD)	*M* (SD)	*M* (SD)
Context willingness to engage in NCFS (Wave 1)					
Relationship partner	1.45 (1.10)	1.32 (0.90)	1.54 (1.17)	1.48 (1.21)	1.43 (1.07)
Dating partner	1.38 (0.92)	1.26 (0.73)	1.49 (1.02)	1.35 (0.95)	1.40 (0.95)
Friend	1.44 (1.07)	1.34 (0.96)	1.59 (1.23)	1.38 (1.01)	1.45 (1.04)
Stranger	1.62 (1.32)	1.43 (1.07)	1.70 (1.38)	1.57 (1.31)	1.82 (1.49)
Ex-partner	1.48 (1.13)	1.38 (1.01)	1.60 (1.27)	1.41 (1.02)	1.55 (1.19)
Context willingness to engage in NCFS (Wave 2)					
Relationship partner	1.55 (1.23)	1.48 (1.13)	1.62 (1.28)	1.60 (1.36)	1.48 (1.12)
Dating partner	1.44 (1.05)	1.38 (0.97)	1.59 (1.21)	1.37 (0.98)	1.40 (0.97)
Friend	1.51 (1.14)	1.50 (1.16)	1.65 (1.26)	1.41 (1.06)	1.46 (1.07)
Stranger	1.60 (1.27)	1.44 (1.07)	1.85 (1.51)	1.41 (1.01)	1.70 (1.40)
Ex-partner	1.50 (1.12)	1.41 (1.00)	1.73 (1.37)	1.32 (0.89)	1.53 (1.14)

#### Instrumental Attitudes Toward Sex

We used the four items with the highest factor loadings from the scale developed by Hendrick and Hendrick (1987) (e.g., “The main goal of sex is that you yourself have a good time”, “Sex is just a game”). Answer options ranged from 1 (*totally disagree*) to 7 (*totally agree*). In both waves, the items formed a unidimensional scale (explained variance > 63%), which had a Cronbach’s alpha of .81 (.84 in Wave 2) (*M* = 3.05, SD = 1.22 in Wave 1; *M* = 3.03, SD = 1.25 in Wave 2).

#### Age and Gender

Age was measured in years and recoded into a dummy variable distinguishing adolescents aged 13–17 [= 0] and emerging adults aged 18–25 [= 1]). Gender was measured by distinguishing female [= 0] and male [= 1] participants. Ten participants (0.51% of the sample) had reported their gender inconsistently over the two waves and received a “missing value” score for gender.

### Data Analysis

To test our hypotheses, we modeled the relationships in AMOS, using a latent construct for pornography use loading on the manifest two items measuring this construct, and manifest items of the five “willingness to engage in NCFS” contexts. The five willingness to engage in NCFS items at Wave 1 were also allowed to predict pornography use at Wave 2. We controlled for age, gender and experience with sexting (i.e., whether participants had ever sent a sexually explicit picture of themselves to others via the internet or their smartphone, coded “yes” [Wave 1 = 7.8%, Wave 2 = 8.2%] or “no”). The latter was included as a control variable as experience with sending sexts would increase the chance of a person ever having received a sext, which could influence their willingness to forward sexts. These manifest control variables were loaded on the exogenous constructs (note that the models did not include age or gender as a control variable when testing these factors as moderators). Endogenous constructs, and disturbance terms of the exogenous constructs, within the same wave were allowed to correlate. Error terms of the same manifest items measuring the latent construct of pornography use were allowed to correlate from Wave 1 to Wave 2. For all main variables (pornography use and the willingness to engage in NCFS variables), constructs at Wave 2 were auto-regressed on the same constructs at Wave 1.

To account for the violation of the normality assumption in our variables, we used the bootstrap method in addition to our parametric tests (Efron & Tibshirani, 1993). We estimated 95% bias-corrected confidence intervals (95% BCI) of the unstandardized estimates on the basis of 1000 bootstrapping samples (due to missing values in the gender variable, BCI’s were calculated on samples of *N* = 1937 each). When the 95% BCI does not include zero, the effect can be assumed to differ significantly from zero and thus refers to a statistically significant relationship. Moreover, to account for family-wise error rate due to testing relationships with five outcome variables in one model, we applied a Bonferroni correction to the *p* values of the regression coefficients, such that only *p* values below .01 were indicative of a significant relationship between pornography use and the willingness to engage in NCFS items. Adequate model fit was based on the following criteria (Byrne, 2010): (1) a *χ*^2^/*df* value below 3.00, (2) a comparative fit index (CFI) of .90 or greater is considered acceptable (.95 or greater indicates good model fit), and (3) a root-mean-square error of approximation (RMSEA) less than .08.

In order to test whether the relationship between pornography use and the willingness to sext depended on instrumental attitudes toward sex, and whether this further differed for adolescent boys and girls and emerging adult men and women, we tested interaction effects using multiple group analyses (cf. Rigdon, Schumacker, & Wothke, 1998) in AMOS. More precisely, we created two groups of high and low instrumental attitudes (at Wave 1) based on a median split (median = 3.00); *N *= 1038 (53.3%) belonged to the low instrumental attitudes group, and *N *=909 (46.7%) belonged to the high instrumental attitudes group. We looked at the significance of the change in model fit when comparing a model where the hypothesized relationships were constrained to be equal between the two groups with a model where the relationships were allowed to vary, for each of the five outcome variables. Each tested relationship (i.e., from pornography use at Wave 1 to the five willingness to engage in NCFS variables at Wave 2) was thus constrained in a separate model, and each of these models was contrasted with the unconstrained model. A significant change in model fit from the unconstrained model to the model with the constrained relationship indicates a significant interaction effect for that particular relationship.

To test further moderation by gender and age, we created eight groups based on people’s scores on instrumental attitudes, their gender and the age group they belonged to (i.e., adolescent females with low vs. high instrumental attitudes, adolescent males with low vs. high instrumental attitudes, emerging adult females with low vs. high instrumental attitudes and emerging adult males with low vs. high instrumental attitudes). We looked at the significance of the change in model fit when comparing a model where the hypothesized relationships were constrained to be equal between the eight groups with a model where the relationships were allowed to vary, for each of the outcome variables. In case of a significant interaction effect, we conducted post hoc analyses where we specifically compared high versus low instrumental attitudes within gender and age groups, using multiple group comparisons.

## Results

### Predicting the Willingness to Engage in Non-Consensual Forwarding of Sexts by Pornography Use and Instrumental Attitudes

Partial correlations between the main variables (i.e., gender, age, pornography use and instrumental sexual attitudes at Wave 1, and the willingness to engage in NCFS outcomes at Wave 2), controlling for experience with sexting, are shown in Table 2. We first tested a model where pornography use at Wave 1 predicted the five types of willingness to engage in NCFS at Wave 2, controlling for experience with sexting, age and gender. The fit of the model was acceptable, *χ*^2^(45, *N* = 1947) = 199.03, *p* < .001, CFI = .99, RMSEA = .042 (90% confidence interval: .036/.048), but the *χ*^2^/*df* value was less than acceptable (= 4.42). We therefore did not interpret the direct relationships between pornography use and the willingness to engage in NCFS further.

**Table 2:** Pearson’s correlations between the main variables, controlling for experience with sexting

	1	2	3	4	5	6	7	8	9
1. Gender (female = 0, male = 1)	1								
2. Age (adolescents = 0, young adults = 1)	− .07*	1							
3. Pornography use (w1)	.40*	.05	1						
4. Instrumental sexual attitudes (w1)	.14*	− .01	.18*	1					
5. NCFS relationship partner (w2)	.01	− .02	.09*	.09*	1				
6. NCFS dating partner (w2)	.07*	− .07*	.11*	.12*	.78*	1			
7. NCFS friends (w2)	.05	− .08*	.12*	.12*	.76*	.75*	1		
8. NCFS stranger (w2)	.14*	− .07*	.24*	.15*	.33*	.55*	.46*	1	
9.NCFS ex-partner (w2)	.13*	− .09*	.16*	.15*	.39*	.59*	.48*	.78*	1

*w1* Wave 1, *w2* Wave 2, *NCFS* willingness to engage in NCFS

*p < .01

To test whether an interaction between pornography use and instrumental attitudes toward sex would predict the willingness to engage in NCFS, we tested two models for groups of low sexual instrumental attitudes and high instrumental attitudes, with moderation effects tested in a multiple group analysis. The unconstrained model in which the low and high instrumental attitudes groups were compared showed good model fit, *χ*^2^(90, *N* = 1947) = 264.08, *p* < .001, CFI = .99, RMSEA = .032 (90% confidence interval: .027/.036), *χ*^2^/*df* = 2.93. Model comparisons showed a, marginally, significant moderation effect of instrumental attitudes for the relationship between pornography use and the willingness to engage in NCFS in the context of a stranger (CMIN = 3.89, *p *= .049). No other moderation effects were found (CMIN < 2.88, *p *>.089). Findings showed that while this relationship was significant among youth with low instrumental attitudes toward sex (*β* = 0.12, *B *=.10, SE=0.03, *p *< .001, 95% BcCI: 0.03/0.18, *R*^2^ = .15), it was stronger among youth with high instrumental attitudes toward sex (*β* = 0.23, *B *=.18, SE=0.03, *p *< .001, 95% BcCI: 0.11/0.25, *R*^2^ = .20).

As can be seen in Table 3, the findings also showed that pornography use was a significant predictor of the willingness to engage in NCFS in the context of a friend and an ex-partner for youth with high instrumental attitudes toward sex, and in the context of a dating partner for youth with low instrumental attitudes toward sex. However, there was no significant moderation effect of instrumental attitudes in these contexts and the explained variance of these outcome variables was much lower compared to the context of a stranger. No significant prediction was found for the willingness to engage in NCFS in the context of a relationship partner.

**Table 3:** Standardized and unstandardized estimates of the prediction of the willingness of non-consensual forwarding of sexts in different contexts (Wave 2) by online pornography use (Wave 1), for individuals with low and high instrumental attitudes toward sex, controlling for gender, age, and experience with sending sexts

	Low instrumental attitudes				High instrumental attitudes			
	*β*	*B* (SE)	95% BcCI	Explained variance (%)	*β*	*B* (SE)	95% BcCI	Explained variance (%)
Context willingness NCFS (Wave 2)								
Dating partner	0.14*	0.09 (0.03)	0.04/0.17	7	0.05	0.03 (0.02)	− 0.02/0.09	8
Relationship partner	0.09	0.07 (0.03)	0.00/0.16	7	0.07	0.05 (0.03)	− 0.01/0.12	11
Friend	0.09	0.07 (0.03)	0.01/0.14	9	0.10*	0.07 (0.03)	0.01/0.13	9
Stranger	0.12**	0.10 (0.03)	0.03/0.17	15	0.23**	0.18 (0.03)	0.11/0.25	20
Ex-partner	0.09	0.06 (0.03)	0.01/0.13	12	0.10*	0.07 (0.03)	0.01/0.13	13

*p <.01; **p < .001

### Interactions with Gender and Age

We looked at the multiple group comparisons comparing the relationships between pornography use and the willingness to engage in NCFS items, between the eight groups based on instrumental attitudes toward sex, gender, and age. The fit of the unconstrained model separated by the 8 groups was good, *χ*^2^(328, *N* = 1947) = 815.65, *p* < .001, CFI = .98, RMSEA = .028 (90% confidence interval: .025/.030), *χ*^2^/*df* = 2.49. Model comparisons showed a significant change in model fit when constraining the relationship between pornography use and the willingness to engage in NCFS in the context of a dating partner (CMIN = 14.50, *p* = .043), friend (CMIN = 18.75, *p* = .009), stranger (CMIN = 43.57, *p* < .001), and ex (CMIN = 25.73, *p* = .001), but not in the context of a relationship partner (CMIN = 7.24, *p* = .404). However, post hoc analyses comparing low and high instrumental attitudes toward sex within each of the 8 groups based on gender and age showed that there was only a significant difference in model fit when constraining the paths for low versus high instrumental attitudes toward sex among male adolescents, for NCFS in the context of a stranger, CMIN = 5.91, *p* = .02. No significant changes in model fit were found for comparing low and high instrumental attitudes toward sex in the other groups for any of the other outcome variables. For male adolescents with low instrumental attitudes toward sex, there was no significant relationship between pornography use and the willingness to engage in NCFS in the context of a stranger, whereas there was a significant relationship for male adolescents with high instrumental attitudes toward sex (see Table 4).

**Table 4:** Standardized and unstandardized estimates for the prediction of the willingness of non-consensual forwarding of sexts in different contexts (Wave 2) by online pornography use (Wave 1), for adolescent boys with low and high instrumental attitudes toward sex, controlling for experience with sending sexts

	Adolescent boys with low instrumental attitudes				Adolescent boys with high instrumental attitudes			
	β	B (SE)	95% BcCI	Explained variance (%)	β	B (SE)	95% BcCI	Explained variance (%)
Context willingness NCFS (Wave 2)								
Dating partner	0.19*	0.13 (0.05)	0.01/0.33	4	0.13	0.09 (0.04)	0.01/0.22	7
Relationship partner	0.17	0.12 (0.05)	− 0.01/0.32	3	0.09	0.06 (0.04)	− 0.03/0.19	7
Friend	0.21*	0.15 (0.05)	0.02/0.30	12	0.19*	0.13 (0.04)	0.04/0.25	11
Stranger	0.12	0.10 (0.06)	− 0.03/0.29	16	0.36**	0.28 (0.05)	0.15/0.42	31
Ex-partner	0.10	0.07 (0.05)	− 0.03/0.23	8	0.22**	0.17 (0.04)	0.06/0.29	18

*p <.01; **p < .001

## Discussion

The present study was one of the first to investigate whether the use of pornography, in interaction with instrumental attitudes toward sex, predicts adolescents’ and young adults’ willingness to forward a sexually explicit image of someone without that person’s consent, in different contexts (i.e., relationship partner, dating partner, friend, stranger, ex-partner). The findings showed that for young people with both low and high levels of instrumental attitudes toward sex, pornography use predicted a higher willingness to engage in the non-consensual forwarding of a sexually explicit image of a stranger. This relationship was stronger for youth with high instrumental attitudes toward sex. Further moderation analyses including gender and age showed that such a prediction occurred predominantly among adolescent boys with high levels of instrumental attitudes toward sex.

### Individual Differences in the Relationship Between Pornography Use and the Willingness to Engage in Non-Consensual Forwarding of Sexts

In line with previous research on individual differences in the effects of pornography (e.g., Kingston et al., 2009; Malamuth & Huppin, 2005; Malamuth et al., 2000), and in particular previous theorizations that the influence of (sexual) media use can be increased (or reduced) by the (lack of) resonance with pre-existing schema’s (Leonhardt et al., 2019; Wright, 2011), we found that the influence of pornography use on the willingness to engage in NCFS was stronger for individuals with high levels of instrumental attitudes toward sex, predominantly in the context of an image by a stranger. These findings point to the importance of taking into account in which context the non-consensual forwarding of sexts occurs. Although NCFS can be considered an instrumental sexual behavior in itself (e.g., a sexual behavior that is focused on fun and excitement rather than intimacy), the present findings show that this is mostly the case for forwarding sexts by strangers. This seems likely because in the contexts of strangers it is easier to look at the sext in an instrumental rather than an intimate way leading to the highest resonance with instrumental attitudes and pornography use. In a context where people know or care about the person in the picture, pornography use and instrumental attitudes toward sex may play less of a role in their willingness to forward such a picture. This is supported by the lack of a significant interaction between pornography use and instrumental attitudes toward sex for any of the other contexts of NCFS, in particular for the willingness to engage in NCFS in the context of a romantic partner.

Furthermore, the finding that adolescent boys with high instrumental attitudes toward were most susceptible to the relationship between pornography use and the willingness to engage in NCFS in the context of a stranger resonates with previous research that showed that pornography use predicted sexual harassment among adolescent boys, not among adolescent girls (Brown & L’Engle, 2009). NCFS may be a type of sexual harassment—or anti-social sexual behavior—that matches particularly well with the lived experience of adolescent boys. As adolescent boys are likely to receive sexts that were originally meant to be private (Fleschler Peskin et al., 2013), they may often encounter a situation in which their willingness to engage in NCFS may lead to actual behavior. Future interventions are thus advised to pay attention to certain risk factors among adolescent boys, such as high pornography use and instrumental attitudes toward sex, in order to reduce or prevent the development of willingness to engage in NCFS, and actual engagement in NCFS.

### Limitations and Suggestions for Future Research

The current study looked at the prediction of the willingness to engage in NCFS by pornography use from a sexual scripting perspective, which is currently the dominant theoretical paradigm for studying the relationships between pornography use and sexual behaviors. One limitation of this study, however, is that we did not look at specific sexual harassment scripts in pornography and its effects on NCFS, which does not allow us to fully test script-based effects of pornography. Although content analyses have shown that sexual harassment scripts are prevalent in the most often watched pornography content (e.g., Cowan & Campbell, 1994; Cowan et al., 1988; Klaassen & Peter, 2015; Monk-Turner & Purcell, 1999; Seto et al., 2001), future research may need to focus more on specific sexual harassment scripts in pornography, and the subsequent internalization of such scripts, to fully test whether and which scripts influence online sexual harassment behaviors such as NCFS.

Relatedly, in line with the sexual scripting perspective, previous research has focused on sexual attitudes, such as instrumental attitudes toward sex, as outcomes and underlying mechanisms of the effects of pornography use rather than moderators (e.g., Peter & Valkenburg, 2010; Vandenbosch & van Oosten, 2018). In fact, given the depiction of sex in an instrumental way in pornography (Brosius et al., 1993; Klaassen & Peter, 2015; Monk-Turner & Purcell, 1999) it seems likely that pornography use increases the willingness to engage in NCFS by increasing instrumental attitudes toward sex. However, the use of a two-wave longitudinal design and the short time-span of 2 months between waves did not allow us to thoroughly test such a mediation model.^1^ Future research using more waves of longer periods of time will be better suited to investigate such indirect effects.

Furthermore, research has pointed at the possibility that sexual attitudes can also be seen as confounds in the relationship between pornography use and sexual behavior, predicting both higher pornography use as well as the sexual behavior. A recent study comparing a sexual-script-based prediction to a confounding prediction showed that the model looking at sexual attitudes as confounds did not hold and that the prediction of sexual behavior by pornography use remains after controlling for confounds such as sexual attitudes (Wright, 2018). That said, other confounding factors, such as peer norms, family functioning, or personality factors such as hypergender orientations or sensation seeking, may influence both pornography use as well as the willingness to engage in NCFS. Future research should thus take into account possible alternative explanations for the relationship between pornography use and (the willingness of) engagement in NCFS to gain a more complete perspective of the antecedents of such behavior.

Another limitation of our research design is that it does not allow us to conclude that pornography use and instrumental attitudes *cause* a higher willingness to engage in NCFS. Such a conclusion would require an experimental design. However, since we are investigating pornography use and anti-social sexual behaviors, such an experimental design would not be possible from an ethical standpoint. Our longitudinal design does allow controlling for earlier levels of willingness to engage in NCFS so that conclusions about changes in such willingness over time, as predicted by pornography use and instrumental attitudes toward sex, can be made. Moreover, the time-span in which we measured such changes was only 2 months. Previous research on sexual health interventions did show that changes in sexual behavior, and in particular sexual risk behavior, can occur in short time spans of only a few months (e.g., Kirby et al., 2007; Oakley et al., 1995). That said, future research is needed to see if the relationships found in the present study hold over longer periods of time.

Since the present study focuses on the willingness to engage in NCFS we cannot draw conclusions on the predictive value of pornography use and instrumental attitudes toward sex for the actual *engagement* in non-consensual forwarding of sexts. However, given that NCFS is an anti-social online behavior, and in fact considered punishable by law, studying this behavior may be problematic. Youth may not report such behavior honestly out of fear of (legal) repercussions which makes it difficult to validly measure such behavior. The willingness to engage in NCFS is a much more subtle measure which is often used for sexual risk behavior and can thus be expected to be more suitable for measuring antisocial behaviors such as NCFS. Moreover, a focus on behavioral willingness fits in a body of research on risk behavior based on the prototype-willingness model (e.g., Gerrard et al., 2008), and the role of media herein (e.g., Boot, Peter, & van Oosten, 2016; Dal Cin et al., 2009; van Oosten, Peter, & Vandenbosch, 2017; van Oosten & Vandenbosch, 2017).

Some of the relationships between pornography use and the willingness to engage in NCFS were significant for one of the instrumental attitudes groups and not for the other (i.e., in the context of a friend, ex-partner or dating partner). However, the lack of significant interaction effects suggests that the resonance effect was not robust for these outcomes. It could be that in such cases, pornography use resonates more with other characteristics that make it more likely that people engage in such anti-social behaviors toward close others. For instance, pornography seems to portray its characters in an objectifying or dehumanized way (Klaassen & Peter, 2015; Monk-Turner & Purcell, 1999; Seto et al., 2001). Such dehumanization, or the denying of human characteristics to another person including the denial of their feelings and suffering (Haslam, 2015), has been associated with sexual harassment (e.g., Marshall & Barbaree, 1991; Rudman & Mescher, 2012). Pornography use could therefore also strengthen individuals pre-existing tendencies to objectify and dehumanize others, including friends and dating partners, and as such increase the willingness to engage in NCFS.

In addition, given the low explained variance for the outcomes of NCFS in the context of a friend, ex- or dating-partner, factors unrelated pornography use are likely to predict the willingness to engage in NCFS in such contexts. For instance, it could be that the peer pressure to “show off”, which was found as a motivation for NCFS in previous research (AP-MTV, 2009; Van Ouytsel et al., 2017), may be more predictive of the willingness to engage in NCFS in the context of a friend or dating partner. Because the peer group is likely to know the friend shown in the sexting image, the pressure to forward this image may be increased. Future research could thus focus on other individual difference variables to see who is more susceptible to the influence of pornography use on the willingness to engage in NCFS, in particular in the context of close others.

### Conclusion

Scholars have started to argue that when preventing negative consequences of sexting we need to focus more on the non-consensual distribution of sexually explicit images, rather than exclusively on the original sender of the image (e.g., Krieger, 2017; Van Ouytsel, Walrave, & Van Gool, 2014). The present study complements such arguments, by showing that such interventions may particularly need to target adolescent boys who are frequent users of pornography and hold pre-existing attitudes of sex as merely “a game” for one’s own pleasure and excitement. This study has thus increased our knowledge on the relationship between pornography use and NCFS, and the need to distinguish between different contexts of NCFS and differential susceptibility of pornography users.

## Electronic supplementary material

Below is the link to the electronic supplementary material.

Supplementary material 1 (DOC 58 kb)

## Footnotes

1 In a previous version of the article, we attempted to test such a mediation model using structural equation modeling, where pornography use at Wave 1 was modeled to predict instrumental attitudes toward sex at Wave 2, which in turn was modeled to predict the willingness to engage in NCFS at Wave 2. Overall, the model fit of the mediation model was worse than the fit of the moderation model presented in the current manuscript. No significant indirect relationships were found, although there was a significant negative relationship between instrumental attitudes toward sex and the willingness to engage in NCFS in the context of a friend, among males only. A supplementary document with these previous results can be obtained upon request from the first author.
