# Supplementary material for: Predicting the Willingness to Engage in Non-Consensual Forwarding of Sexts: The Role of Pornography and Instrumental Notions of Sex
Source: Arch Sex Behav. 2020 Jan 31;49(4):1121–32. doi: 10.1007/s10508-019-01580-2 (PMC7145774; doi:10.1007/s10508-019-01580-2)
Supplement: Supplementary file 1 — Supplementary material 1 (DOC 58 kb) [file 10508_2019_1580_MOESM1_ESM.doc]

**Measures**

**Exposure to Online Pornography.** Participants were asked how often in the previous two months they had intentionally looked at sexually explicit content (i.e., pornographic material, not nudity) on their computer, either online or offline (i.e., downloaded material) (cf. Peter & Valkenburg, 2009, 2010). Sexually explicit content was specified as (a) pictures with clearly exposed genitals, (b) movies with clearly exposed genitals, (c) pictures in which people were having sex, and (d) movies in which people were having sex. For each type of sexual content, the response categories were 1 (*several times a day*), 2 (*every day*), 3 (*several times a week*), 4 (*once a week*), 5 (*1-3 times a month*), 6 (*less than once a month*), and 7 (*never*). Items were recoded such that higher scores indicated more frequent use of online pornography. In both waves, the items formed a unidimensional scale (explained variance > 85%), which had a Cronbach’s alpha of .94 (*M* = 1.86, *SD* = 1.42 in wave 1; *M* = 1.90, *SD* = 1.44 in wave 2).

**Willingness to Engage in Non-Consensual Forwarding of Sexts.** The measure consisted of two parts, the first measuring whether respondents had ever engaged in NCFS (which we also used as an outcome variable in additional analyses, see the Results), and the next part measuring their willingness to engage in that behavior. The first part was introduced by the following explanation (the adult version is shown between brackets): “Some boys (men) and girls (women) will send a picture of themselves to someone else via the internet or their smartphone in which they are naked or almost naked. Sometimes teens (people) forward such a picture to others without asking the person in the picture for permission.” After being asked about whether they had ever engaged in this behavior themselves (“yes” or “no”), participants were presented with the following scenario for the second part of the measure: “Imagine you received an image or video fragment on your phone or online that shows a person who is naked or almost naked. After you received it, you could forward this image or video to others without the sender being aware of it. Try to picture how likely it is that you would forward this image or video when it shows the following person: a) the person that you are in a romantic relationship with; b) the person that you are dating; c) a friend; d) someone you do not know; e) your ex.” For each of the five persons that were mentioned the answer options ranged from 1 *“very likely”* to 7 *“very unlikely”.* Answers were recoded so that higher scores indicated a higher willingness to engage in NCFS. The five items were analyzed as separate outcome variables (see Table 1 for the properties of both the engagement in NCFS and the willingness to engage in NCFS measure).

**Sexual Objectification of Women.** Four items from Peter and Valkenburg’s adjusted version (2007) of Ward’s (2002) scale were used to measure participants’ endorsement of sexual objectification of women (e.g., “An attractive woman should expect sexual advances”, “It’s okay if a man is only interested in a woman for her body”). Answer options ranged from 1 (*totally disagree*) to 7 (*totally agree*). In both waves, the items formed a unidimensional scale (explained variance > 57%), which had a Cronbach’s alpha of .74 (.76 in wave 2) (*M* = 3.28, *SD* = 1.21 in wave 1; *M* = 3.26, *SD* = 1.21 in wave 2).

**Instrumental Attitudes towards Sex.** We used the four items with the highest factor loadings from the scale developed by Hendrick and Hendrick (1987) (e.g., “The main goal of sex is that you yourself have a good time”, “Sex is just a game”). Answer options ranged from 1 (*totally disagree*) to 7 (*totally agree*). In both waves, the items formed a unidimensional scale (explained variance > 63%), which had a Cronbach’s alpha of .81 (.84 in wave 2) (*M* = 3.05, *SD* = 1.22 in wave 1; *M* = 3.03, *SD* = 1.25 in wave 2).

**Data Analysis**

To test our hypotheses, we modelled the relationships in AMOS, using latent constructs for pornography use and the sexual attitude variables, loading on the manifest items measuring each construct, and on the manifest items for the five willingness to engage in NCFS outcp,e variables. We controlled for the manifest variables of age (dummy variable distinguishing adolescents aged 13-17 [= 0] and emerging adults aged 18-25 [=1]) and gender (dummy variable distinguishing female [= 0] and male [=1] participants) by loading them on the exogenous constructs (note that the models did not include age or gender as a control variable when testing each of these factors as moderators). Endogenous constructs, and disturbance terms of the exogenous constructs, within the same wave were allowed to correlate. Error terms of the same manifest items measuring the latent constructs were allowed to correlate from wave 1 to wave 2. For all main variables (pornography use, sexual attitudes and the willingness to engage in NCFS variables), constructs at wave 2 were auto regressed on the same constructs at wave 1.

To account for the violation of the normality assumption in our variables, we used the bootstrap method in addition to our parametric tests (Efron & Tibshirani, 1993). We estimated 95% bias-corrected confidence intervals (95% BCI) of the standardized estimates on the basis of 1,000 bootstrapping samples (N = 1,947 each). When the 95% BCI does not include zero, the effect can be assumed to differ significantly from zero, and thus refers to a statistically significant relationship.

In order to test if our hypothesized relationships differed for adolescents and emerging adults, or for males and females, we tested interaction effects using multiple group analyses (cf. Rigdon, Schumacker, & Wothke, 1998) in AMOS. More precisely, we looked at the significance of the change in model fit when comparing a model where one of the hypothesized relationships was constrained to be equal between groups (either adolescents vs. emerging adults, or males versus females) with a model where this relationship was allowed to vary. Each tested relationship (i.e., from pornography use at wave 1 to the two sexual attitudes at wave 2, H2a&b, and from the two sexual attitudes at wave 2 to the five willingness to engage in NCFS variables at wave 2, H3a&b) was thus constrained in a separate model, and each of these models was contrasted with the unconstrained model. A significant change in model fit from the unconstrained model to the model with the constrained relationship indicates a significant interaction effect for that particular relationship.

**Additional Analysis: Engagement in NCFS.** Despite the small percentage of youth who had ever engaged in NCFS in our sample, we also tested whether pornography use at wave 1 (H1), and the sexual attitudes at wave 2 (H3a/b), could predict whether a person would have engaged in NCFS at wave 2, in additional analyses in AMOS. The models were built in a similar way as for the main analyses, with the only difference that the five manifest variables for willingness to engage in NCFS were replaced with one manifest item for engagement in NCFS (1= ever engaged in NCFS, 0 = never engaged in NCFS). To account for the violation of the normality assumption in our variables and, most importantly, the dichotomous nature of our main outcome variable (NCFS), Bayesian estimation was used (for more information see Byrne, 2010). The parameter estimations were considered adequate when the model reached a convergence statistic (C.S.) of 1.001 and the Bayesian standard error for each parameter estimate was lower than .02 (Byrne, 2010). Bayesian confidence intervals (95% CI) that do not include zero are interpreted as statistically significant relationships.

**Results**

**Predicting the Willingness to Engage in NCFS**

To test H1, we tested a model where pornography use at wave 1 predicted the five types of willingness to engage in NCFS at wave 2, controlling for age and gender. Also, willingness to engage in NCFS at wave 1 was allowed to predict pornography use at wave 2 as the Sexual Behavior Sequence theory would also expect the sexual arousal triggered by NCFS may drive users to consume more pornography. The fit of the model was less than acceptable, χ² (107, N = 1,947) = 2124.50, *p* < .001, CFI = .94, RMSEA = .099 (90% confidence interval: .095/.103), χ²/df = 20.02, meaning that the data does not represent the relationships in the population well. The data showed that pornography use at wave 1 significantly predicted the willingness to engage in NCFS in all five contexts (see Table 2), although caution is warranted given the low fit of the model to the data.

To test H2a/b and H3a/b, we tested a model in which the latent constructs of the sexual attitudes were added to the model. The sexual attitudes at wave 2 were regressed on pornography use at wave 1, and the willingness to engage in NCFS items at wave 2 were regressed on the sexual attitudes at wave 2. Indirect relationships were tested using bootstrapping. The fit of the indirect model was acceptable, χ² (496, N = 1,947) = 3730.28, *p* < .001, CFI = .94, RMSEA = .058 (90% confidence interval: .056/.060), χ²/df = 7.52. Pornography use at wave 1 significantly predicted sexual objectification of women at wave 2, *β* = .05, *B* = .04, *SE* = .02, *p* = .033, and instrumental attitudes towards sex at wave 2, *β* = .06, *B* = .05, *SE* = .02, *p* = .012, confirming H2a and H2b. As shown in Table 3, only sexual objectification of women (at wave 2), and not instrumental attitudes towards sex (at wave 2), significantly predicted the willingness to engage in NCFS at wave 2, supporting H3a only. Instrumental attitudes towards sex (at wave 2) seemed to negatively predict the willingness to engage in NCFS in the context of a romantic relationship partner or a friend, but this was not significant with bootstrapping. The indirect relationship between pornography use (wave 1) and the willingness to engage in NCFS (wave 2) through sexual attitudes at wave 2 was not significant.

**Moderation of Age and Gender**

We found significant moderation of age only for the relationship between sexual objectification of women (wave 2) and the willingness to engage in NCFS in the context of a stranger (wave 2), Δχ² = 5.16, *p =* 0.023. This relationship was stronger for adolescents, β = 0.39, *B =* .43, *SE =* 0.06, *p* < .001, 95% BcCI: 0.26/0.66, compared to emerging adults, β = 0.22, *B =* .23, *SE =* 0.05, *p* < .001, 95% BcCI: 0.11/0.39, but significant among both age groups. The indirect relationships between pornography use at wave 1 and willingness to engage in NCFS at wave 2, via sexual attitudes at wave 2, were significant among adolescents for the context of a romantic relationship partner , *B =* 0.02 *, SE =*.01, *p =* .034, dating partner, *B =* 0.02, *SE =*.01, *p =* .031, friend, *B =* 0.02, *SE =*.02, *p =* .043, ex, *B =* 0.02, *SE =*.01, *p =* .047, and marginally significant for strangers, *B =* 0.02, *SE =*.02, *p =* .066. The indirect relationships were not significant among emerging adults, with p-values ranging between .616 and .856.

As for the moderation of gender, we found significant differences between males and females (Δχ² ranging from 4.68 to 18.04) in the relationships between the sexual attitudes – both instrumental attitudes towards sex and sexual objectification of women – at wave 2 and the willingness to engage in NCFS at wave 2 in all contexts except for relationship partner (Δχ² of 2.19 and lower). The relationships between sexual objectification of women and the willingness to engage in NCFS were stronger for males compared to females, but significant among both groups. Among males, but not females, there were negative relationships between instrumental attitudes towards sex and the willingness to engage in NCFS, but these relationships were not significant with bootstrapping except for in the context of a picture of a friend (*β* = -0.16, B = -0.17 , SE = 0.05, *p* < .01, 95% BcCI: -0.64/-0.002). The indirect relationships between pornography use at wave 1 and willingness to engage in NCFS at wave 2, via sexual attitudes at wave 2, was not significant for both males and females (although for males they were marginally significant with *p* values ranging between .06 and .08).

**Additional Analyses: Engagement in NCFS**

For H1, a model where pornography use at wave 1 predicted NCFS at wave 2 (and NCFS at wave 1 predicted pornography use at wave 2), controlling for age and gender, was tested. The model met the reliability conditions for Bayesian estimation with a C.S. value of 1.0018 and all Bayesian *SE’s* of parameters < .02. Pornography use at wave 1 significantly predicted NCFS in wave 2 (standardized direct effect mean = .11, 95% CI: .016/.208). For H3a/b, the prediction of NCFS (wave 2) by sexual attitudes (at wave 2) was added to the model. The model met the reliability conditions for Bayesian estimation with a C.S. value of 1.0018 and all Bayesian *SE’s* of parameters < .02. In contrast to our expectations, and to our analyses for willingness to engage in NCFS, neither instrumental attitudes in wave 2 (standardized direct effect mean = .008, 95% CI: -.135/.141) nor sexual objectification in wave 2 (standardized direct effect mean = .101, 95% CI: -.048/.246) significantly predicted the NCFS in wave 2. The hypotheses for our indirect relationships were thus not supported for the prediction of engagement in NCFS.

To test for moderation effects, the Deviance Information Criterion statistic was used to compare model fits; a lower DIC score means a better fit of the model to the data. According to Spiegelhalter, Best, Carlin, and van der Linde (2002), changes in the DIC larger than 3 receive additional consideration. For our model comparison tests, a DIC change >3 may mean the constraining of a path may substantially lower the model fit and thus indicate that age or gender moderate the relationship. However, when the constrained model has a superior fit, the change in DIC suggests that especially a more parsimonious model seems more appropriate and thus that there is no moderation effect. The analyses resulted in DIC changes of lower than 3, or better model fit for the constrained models, which means the hypothesized relationships for engagement in NCFS were not moderated by age or gender.

References

Efron, B., & Tibshirani, R. J. (1993). *An introduction to the bootstrap*. Boca Raton, FL: Chapman & Hall.

Hendrick, S., & Hendrick, C. (1987). Multidimensionality of sexual attitudes. *Journal of Sex Research*, *23*, 502–526. doi:10.1080/00224498709551387

Peter, J., & Valkenburg, P. M. (2007). Adolescents’ exposure to a sexualized media environment and their notions of women as sex objects. *Sex Roles*, *56*, 381–395. doi:10.1007/s11199-006-9176-y

Peter, J., & Valkenburg, P. M. (2009). Adolescents’ exposure to sexually explicit Internet material and notions of women as sex objects: Assessing causality and underlying processes. *Journal of Communication*, *59*, 407–433. doi:10.1111/j.1460-2466.2009.01422.x

Peter, J., & Valkenburg, P. M. (2010). Processes underlying the effects of adolescents’ use of sexually explicit Internet material: The role of perceived realism. *Communication Research*, *37*, 375–399. [doi:10.1177/0093650210362464](http://doi.org/10.1177/0093650210362464)

Rigdon, E. E., Schumacker, R. E., & Wothke, W. (1998). A comparative review of interaction and nonlinear modeling. In R. E. Schumacker & G. A. Marcoulides (Eds.), *Interaction and nonlinear effects in structural equation modeling* (pp. 1-16). Mahwah, NJ: Lawrence Erlbaum.

Ward, L. M. (2002). Does television exposure affect emerging adults’ attitudes and assumptions about sexual relationships? Correlational and experimental confirmation. *Journal of Youth and Adolescence*, *31*, 1–15. [doi:10.1023/A:1014068031532](http://doi.org/10.1023/A:1014068031532)
